# Expression of a2 Vacuolar ATPase in Spermatozoa is Associated with Semen Quality and Chemokine-Cytokine Profiles in Infertile Men

**DOI:** 10.1371/journal.pone.0070470

**Published:** 2013-07-30

**Authors:** Kuniaki Ota, Mukesh Kumar Jaiswal, Sivakumar Ramu, Rajasinjham Jeyendran, Joanne Kwak-Kim, Alice Gilman-Sachs, Kenneth D. Beaman

**Affiliations:** 1 Department of Microbiology and Immunology, Rosalind Franklin University of Medicine and Science, North Chicago, Illinois, United States of America; 2 Department of Obstetrics and Gynecology, Rosalind Franklin University of Medicine and Science, Vernon Hills, Illinois, United States of America; 3 Andrology Laboratory Services, Inc., Chicago, Illinois, United States of America; University of Leuven, Rega Institute, Belgium

## Abstract

**Background:**

A number of laboratory tests have been developed to determine properties of spermatozoa quality but few have been adopted into routine clinical use in place of the WHO semen analysis. We investigated whether Atp6v0a2 (a2 isoform of vacuolar ATPase) is associated with abnormal semen quality and changes in chemokine-cytokine profiles in infertile men.

**Patients and Methods:**

Semen samples were collected from 35 healthy donors and 35 infertile men at the Andrology laboratory from August 2011 to June 2012. The levels of Atp6v0a2 mRNA and protein, and its localization in spermatozoa were determined. a2NTD (the N-terminal portion of Atp6v0a2) and secreted chemokine-cytokine profiles in seminal fluid were measured.

**Results:**

Atp6v0a2 protein (P<0.05) and mRNA (P<0.05) in spermatozoa from infertile men were significantly lower than those from fertile men. Fluorescent microscopy revealed that Atp6v0a2 is mainly expressed in the acrosomal region. Infertile men’s seminal fluid had significantly lower G-CSF (P<0.01), GM-CSF (P<0.01), MCP-1 (P<0.05), MIP-1α (P<0.01) and TGF-β1 (P<0.01) levels when compared to the seminal fluid from fertile men. Seminal fluid a2NTD levels were significantly correlated with G-CSF (P<0.01), GM-CSF (P<0.01), MCP-1 (P<0.05), MIP-1α (P<0.01) and TGF-β1 (P<0.01) which are key molecules during the onset of pregnancy.

**Conclusion:**

These results suggested that a critical level of Atp6v0a2 is required for the fertile spermatozoa and its decreased level in spermatozoa could be used to predict male infertility. This study provides a possibility that Atp6v0a2 could be potentially used as a diagnostic marker for the evaluation of male infertility.

## Introduction

The vacuolar (H+)-ATPase (V-ATPase) is a multi-subunit enzyme that couples ATP hydrolysis to the pumping of protons across plasma membranes. It is ubiquitously expressed in eukaryotic cells, where it participates in the acidification of highly differentiated organelles, including the Golgi apparatus, lysosomes, endosomes, and secretory vesicles [1], [2], [3]. In addition, the V-ATPase is also found at high density in the plasma membrane of specialized epithelial cells that are involved in active proton transport and pH regulation of extracellular compartments. Those plasma membrane V-ATPases have important roles in such processes as renal acidification [3], bone resorption [4] or spermatozoa capacitation [5]. In a murine study, V-ATPases in the apical membrane of epididymal cells, which are also controlled by reversible endocytosis and exocytosis, are required for spermatozoa maturation, viability and pH homeostasis [5]. In addition, the a2 isoform of V-ATPase (Atp6v0a2) is located specifically in the acrosomal membrane of murine spermatozoa to regulate an acidic intra-acrosomal pH, which is necessary for processing protease zymogen, essential for fertilization [6]. In agreement with this previous study, Atp6v0a2 was highly expressed in the acrosomal region of the capacitated murine spermatozoa but not detected in non-capacitated spermatozoa from the caudal epididymis [7]. This study provided a new insight into a possible association of Atp6v0a2 with male infertility.

Although seminal fluid has been conventionally viewed as transport media for spermatozoa traversing the female reproductive tissues, it is now known to have broader biological actions in regulating female fertility. Seminal fluid contains a complex array of cytokines, chemokines, and other bioactive molecules [8], [9]. Seminal fluid induces pro-inflammatory cytokines and chemokines such as GM-CSF, IL-6, IL-8, MCP-1, MIP-3α, and IL-1α in the female reproductive tract [10]. Particularly, IL-1 has a potential role in the regulation of blastocyst implantation during early pregnancy [11], . IL-1 enhances V-ATPase activity, and increased level of IL-1 may feedback to down regulate the innate immune response, which is essential for implantation [13]. We have shown that Atp6v0a2 can regulate IL-1β as well as IL-1α with little or no subsequent increase in TNF-α secretion [14], [15]. In addition, sperm capacitation induces the release of a2NTD, which is N-terminal portion from Atp6v0a2. We have shown that a2NTD induces maternal inflammatory cytokines such as LIF, IL-1β, TNF-α and MIP-1α, and exposure of the uterus to spermatozoa accompanied by seminal fluid enhances pregnancy success rate in the murine model [7].

In approximately 30% of couples, male factor infertility is the only cause of infertility, and in another 20% to 30% of couples, it is a contributing factor for their infertility [16]. Semen analysis is the most commonly used diagnostic tool for male infertility. Recently, the World Health Organization (WHO) has issued standards for abnormal semen analysis in 2010 [17]. However, these standards are not quantitative and do not identify abnormal parameters related to the underlying causes of infertility. To issue these standards, semen obtained only from fertile men were used, and there were no “threshold values” for spermatozoa concentration, motility, and morphology to differentiate men as subfertile, of indeterminate fertility, or fertile [18]. Thus, none of these parameters can predict the fertile capacity of spermatozoa or pregnancy outcome with a great deal of confidence. Unfortunately, most clinical laboratories still rely on semen analysis only based on standards to determine plan of care. Indeed, even with techniques such as IVF or IVF with intracytoplasmic spermatozoa injection (ICSI), pregnancy success rates are still remain at 25–30% [19]. This could be partly related to lack of our understanding of the molecular pathology of spermatozoa and semen. Therefore, if a new biomarker could be associated with spermatozoa from infertile men, this would provide a better method to predict fertilization capacity of spermatozoa and pregnancy outcome.

Based on the findings from our lab and others, in this study, we aim to investigate Atp6v0a2 expression and localization in human spermatozoa, and examine the possibility of Atp6v0a2 as a useful biomarker for male factor infertility.

## Materials and Methods

### Study Population and Semen Analysis

We evaluated 35 spermatozoa samples from infertile men aged who were diagnosed with male infertility and undergoing assisted reproduction techniques (ART) such as IVF/ICSI at the Andrology Lab, Chicago, IL, USA. Duration of infertility was at least 12 months or more, and azospermic men were excluded from the study. Additionally, semen samples were obtained from 35 fertile donors who had at least one or more live born infant before they had vasectomy for male sterilization. All the collected samples were evaluated at Department of Microbiology and Immunology, the Chicago Medical School at Rosalind Franklin University of Medicine and Science, IL, USA. The study was approved by the IRB of the Rosalind Franklin University of Medicine and Science. Written informed consent was obtained from all study patients and controls prior to enter the study, which was approved by the local Institutional Review Board (IRB). Before the implementation of the ART, semen samples were collected in sterile containers by masturbation after 3–5 days of sexual abstinence. After liquefaction of the semen at room temperature (22°C) for 30 minutes, the samples were assessed for ejaculate volume, spermatozoa concentration, motility and morphology. Morphology of spermatozoa was studied using Kruger’s strict criteria [20]. The samples were classified according to 2010 WHO semen analysis guidelines [17]. An aliquot of each semen sample from normal fertile donors and infertile men was collected by centrifugation at 400×*g* for 5 minutes to obtain spermatozoa-free seminal fluid as previous reported [21] and then frozen at −80°C until analysis was made. Absence of spermatozoa was verified by H&E staining of seminal fluid. The spermatozoa pellet from each infertile man or normal fertile donor was used to determine the levels of Atp6v0a2 by the Real-time PCR and Western blot.

### Isolation of Motile and Immotile Spermatozoa from Semen by Density Centrifugation

PureSperm gradients 40% and 80% (Nidacon international, Gothenburg, Sweden) were used for the separation of motile and immotile spermatozoa. After 20 minutes of centrifugation at 300×g, motile spermatozoa were harvested from the lowest layer and immotile spermatozoa from the next layer up. After purification, the motility was ≥95% in the motile fraction and <10% in the immotile fraction.

### Real-time Quantitative RT-PCR

Total RNA was isolated from the spermatozoa pellet recovered from normal fertile donors and infertile men by using the RNeasy mini kit (Qiagen, Valencia, CA, USA) with on-column DNase digestion using an RNase-free DNase set (Qiagen, Valencia, CA, USA) according to the manufacturer’s instructions. RNA was stored at −80°C until further use. The cDNA was generated from 1 µg of total cellular RNA by reverse transcription at 50°C for 60 min using random hexamer primer of transcription first strand cDNA synthesis kit (Roche Diagnostics GmBH). Duplex real time-PCR was performed by TaqMan analysis using the Applied Biosystems Step One Real-time PCR system for *Atp6v0a2* and a housekeeping gene *β-actin*. The gene expression of target gene was measured and normalized to the *β-actin* housekeeping gene. Real-time PCR was performed using universal PCR master mix reagent (Applied Biosystems, Foster city, CA) according to the manufacturer’s instructions. The prevalidated Taqman gene expression assays for *Atp6v0a2* (Hs00429389_m1) and β-actin (Hs01060665_g1) for TaqMan assays were obtained from Applied Biosystems.

### ELISA for a2NTD

Recombinant N-terminal portion from Atp6v0a2 (a2NTD) was expressed and purified from *Escherichia coli* and subjected to endotoxin removal column chromatography (Proteome Resources, Aurora, CO, USA). The levels of a2NTD were measured in spermatozoa free-seminal fluid recovered from normal fertile donors and infertile men by using sandwich enzyme-linked immunosorbent assay (ELISA). First, chicken anti-a2NTD (Proteome Resources, Aurora, CO, USA) was added to polystyrene trays overnight. After discarding this antibody, wells were washed with PBST three times. 100 µl of spermatozoa free-seminal fluid samples were added to each well with 25 µl of incubation buffer and incubated overnight at 4°C. Each well was washed with wash buffer three times. Then incubated with 50 µl of biotinylated chicken anti-a2NTD at RT for 2 h. Wells were washed with wash buffer four times. 100 µl of streptavidin-HRP working solution was added to each well and incubated at room temperature for 30 min. Then the wells were washed with wash buffer four times and incubated with 100 µl of stabilized chromogen solution (R&D system, Minneapolis, MN, USA) at RT for 30 min in the dark. The reaction was stopped with 100 µl of stop solution, and then the absorbance was read at 450 nm. The concentration of a2NTD was measured by plotting linear curve.

### Western Blotting

Spermatozoa pellet from normal fertile donors and infertile men were washed two-times with PBS. Equal amounts of protein (50 µg) from total spermatozoa lysates were resolved on 4–20% SDS-PAGE and blotted onto PVDF transfer membranes. The membranes were blocked at room temperature for 1 hour in protein free blocking TBS-T buffer. The blots were then probed with rabbit anti-Atp6v0a2 polyclonal antibody (3 µg/ml) (anti-a2V) (SAB2100187; Sigma-Aldrich, St Louis, MO, USA) or mouse anti-human β-actin (Sigma-Aldrich, St Louis, MO, USA) followed by donkey anti-rabbit IRDye-800CW (1∶20,000), and goat anti-mouse IRDye-680CW (LI-COR Biosciences, Lincoln, Nebraska, USA) respectively. Fluorescent blots were imaged on the Odyssey Infrared Imaging System (LI-COR Biosciences, Lincoln, Nebraska, USA).

### Immunofluorescence Staining

Motile and immotile spermatozoa were isolated as described above and then fixed in 4% paraformaldehyde for 20 min RT and then air-dried onto slides. Spermatozoa were permeabilized and blocked with 0.1% Triton X-100 and 3% BSA for 10 min at room temperature, then washed with PBS. Slides were then incubated with primary anti-a2V antibody (1∶50), diluted in 1% BSA overnight at 4°C. Slides were washed with PBST three times for 10 min each, then incubated with donkey anti-rabbit IgG secondary antibody conjugated with FITC (1∶40, 30 min, at 37°C) (sc-2090; Santa Cruz Biotechnology, Santa Cruz, CA, USA), then washed and mounted in Vectashield with DAPI (Vector Laboratories, Burlingame, CA, USA) and stored at 4°C in the dark. The stained slides were viewed under a fluorescent microscope (Nikon Eclipse 80i; Nikon Inc, Tokyo, Japan). The fluorescence of FITC was monitored using a B-2 filter with a 495 nm band pass barrier filter.

### Multiplex Assays

All procedures were following manufacturer’s instructions (Milliplex MAP kit, Millipore Crop, Billerica, MA, USA) and samples were analyzed on the MAGPIX instrument. The concentration of the analytes was determined in spermatozoa free-seminal fluid recovered from normal fertile donors and infertile men by using the Bio-Plex Manager version 5.0 and MAGPIX xPONENT software, respectively. The assays were run in triplicate to confirm the results. Analytes were normalized to total protein concentration which was estimated with a Bradford assay, a colorimetric protein assay. Twelve analytes were determined: IL-1β, IL-2, IL-4, IL-6, IL-10, IL-12, IFN-γ, monocyte chemo-attractant protein 1(MCP-1), macrophage inflammatory protein-1α (MIP-1α), TNF-α, granulocyte colony-stimulating factor (G-CSF), and granulocyte-macrophage colony-stimulating factor (GM-CSF).

### TGF-β ELISA

TGF-β level were analyzed in spermatozoa free-seminal fluid recovered from normal fertile donors and infertile men by using Quantikine TGF-β ELISA kit (R&D systems, Minneapolis, MN) in accordance with the protocol specified by the manufacturer.

### Statistical Analysis

A detailed statistical analysis was performed using SPSS version 20. Mann-Whitney U-test was applied to compare Atp6v0a2 mRNA and protein levels, and student t-test for the comparison of spermatozoa parameters, seminal fluid cytokine and chemokine levels of the two groups. Spearman’s test was used to determine correlations between a2NTD and cytokine and chemokine levels. A probability of <0.05 and <0.01 was considered to be significantly different.

## Results

### Characteristics of Study Population

The semen profiles of infertile men and normal fertile donors are shown in Table 1. Semen from controls had no abnormal parameters. On the other hand, semen from study group had at least one or more abnormal findings in spermatozoa volume, concentration, motility or morphology. Semen volume (P<0.01), sperm concentration (P<0.05) and sperm motility (P<0.01) of study group were significantly decreased in the infertile men when compared to the normal fertile donors.

**Table 1:** Characteristics of the semen samples from infertile men and normal fertile donors (n = 35 each).

	Infertile men	Normal fertile donors
Semen volume (ml)	1.5±0.1	3.2±0.3**
Spermatozoa concentration (×10*6/ml)	62.4±35.8	80.0±28.8*
Motility (%)	24.0±16.1	60.2±13.7**

Note: data presented as means ±standard deviation.

* P<0.05;

** P<0.01 with respect to normal fertile donors.

### Atp6v0a2 mRNA and Protein Expression in Human Spermatozoa

**Figure 1:**
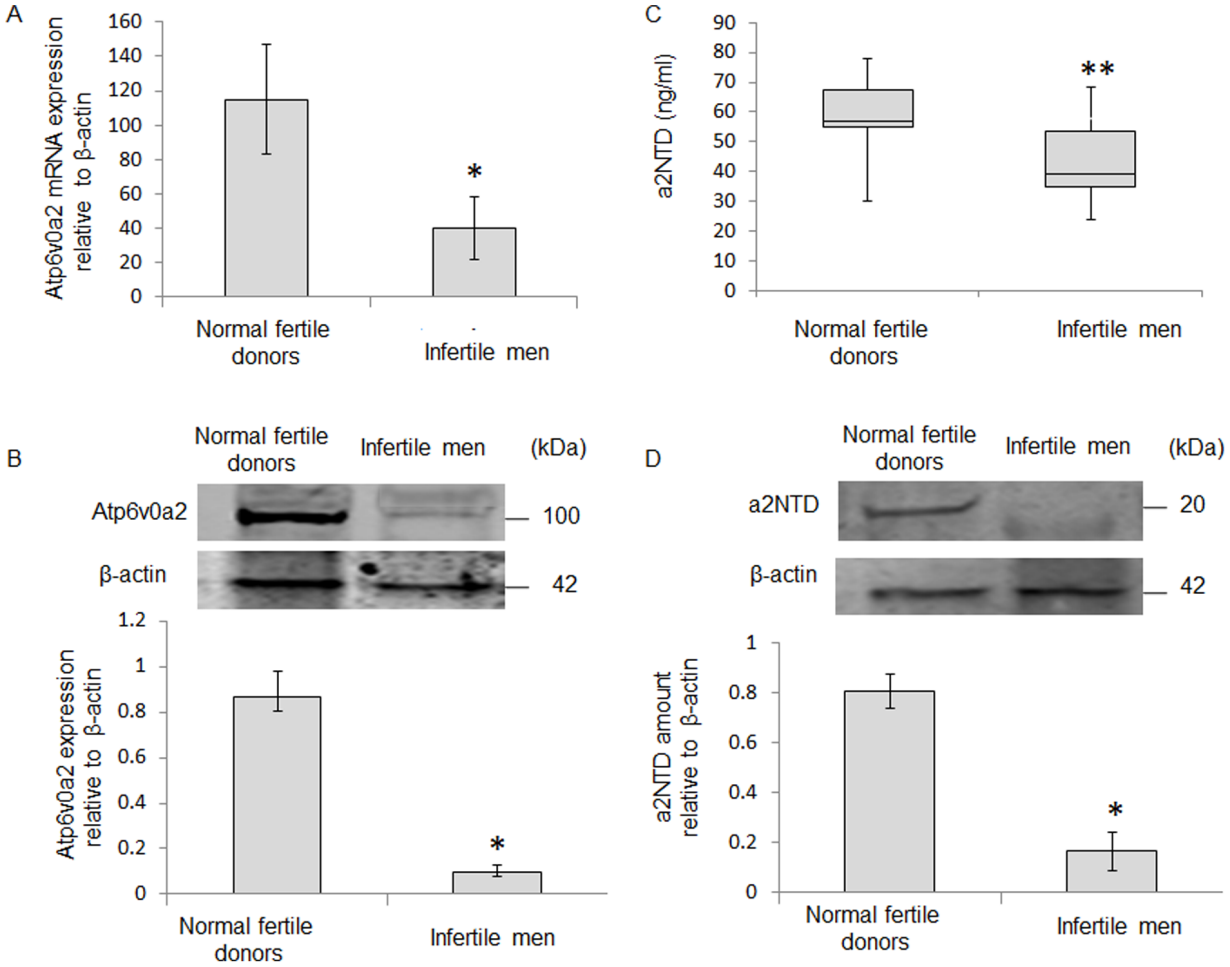
Atp6v0a2 is lower in spermatozoa of infertile men. (A) Atp6v0a2 mRNA expression in spermatozoa was measured by Real-time PCR and gene expression was normalized to β-actin mRNA. Atp6v0a2 mRNA expression in spermatozoa of normal fertile donors was significantly higher than infertile men (P<0.05, n = 18 each); (B) The western blot data indicate the higher level of Atp6v0a2 protein in spermatozoa of normal fertile donors than infertile male. The western blot is a representative of five different experiments. The bar diagram shows the quantification of Atp6v0a2 protein expression, as determined by densitometry of the Atp6v0a2 protein band and normalized to β-actin. Atp6v0a2 protein expression of spermatozoa was significantly higher in normal fertile donors as compared to infertile men (P<0.05, n = 5 each). (C) The level of a2NTD in spermatozoa-free seminal fluid of normal fertile donors was significantly higher than infertile men (P<0.01, n = 20 each). (D) Western blot analysis shows a higher level of a2NTD in spermatozoa-free seminal fluid of normal fertile donors than infertile men. β-actin served as a loading control. The bar diagram shows relative quantification of a2NTD peptide by densitometry analysis and normalized by β-actin. Seminal fluid a2NTD level was significantly higher in normal fertile donors than infertile men (P<0.05, n = 6). *P<0.05; **P<0.01 with respect to normal fertile donors.

The Atp6v0a2 gene expression was analyzed by real time PCR in the spermatozoa recovered from infertile men and normal fertile donors (n = 18 each). The level of Atp6v0a mRNA was significantly decreased in the spermatozoa recovered from infertile men when compared to the normal fertile donors (P<0.05) (Fig. 1A). Western blotting analysis was confirmed that Atp6v0a2 protein was significantly lower (P<0.05, n = 5) in the spermatozoa recovered from infertile men when compared to the normal fertile donors (Fig. 1B).

### Levels of Secreted a2NTD in Seminal Fluid

The concentration of secreted a2NTD was measured in the spermatozoa-free seminal fluid recovered from infertile men and normal fertile donors. Secreted a2NTD protein was significantly higher in normal fertile donors group (57.9±11.0 ng/ml, n = 20) when compared to infertile men group (45.7±16.3 ng/ml, n = 20; P<0.01) (Fig. 1C). Similarly, western blot analysis of spermatozoa free-seminal fluid with anti-a2NTD shows that a2NTD was detected in ∼20 kDa band, and its level was significantly higher in normal fertile donors group compared to infertile men group (P<0.05, n = 6 each) (Fig. 1D).

### Seminal Fluid Cytokine and Chemokine Profiles

**Figure 2:**
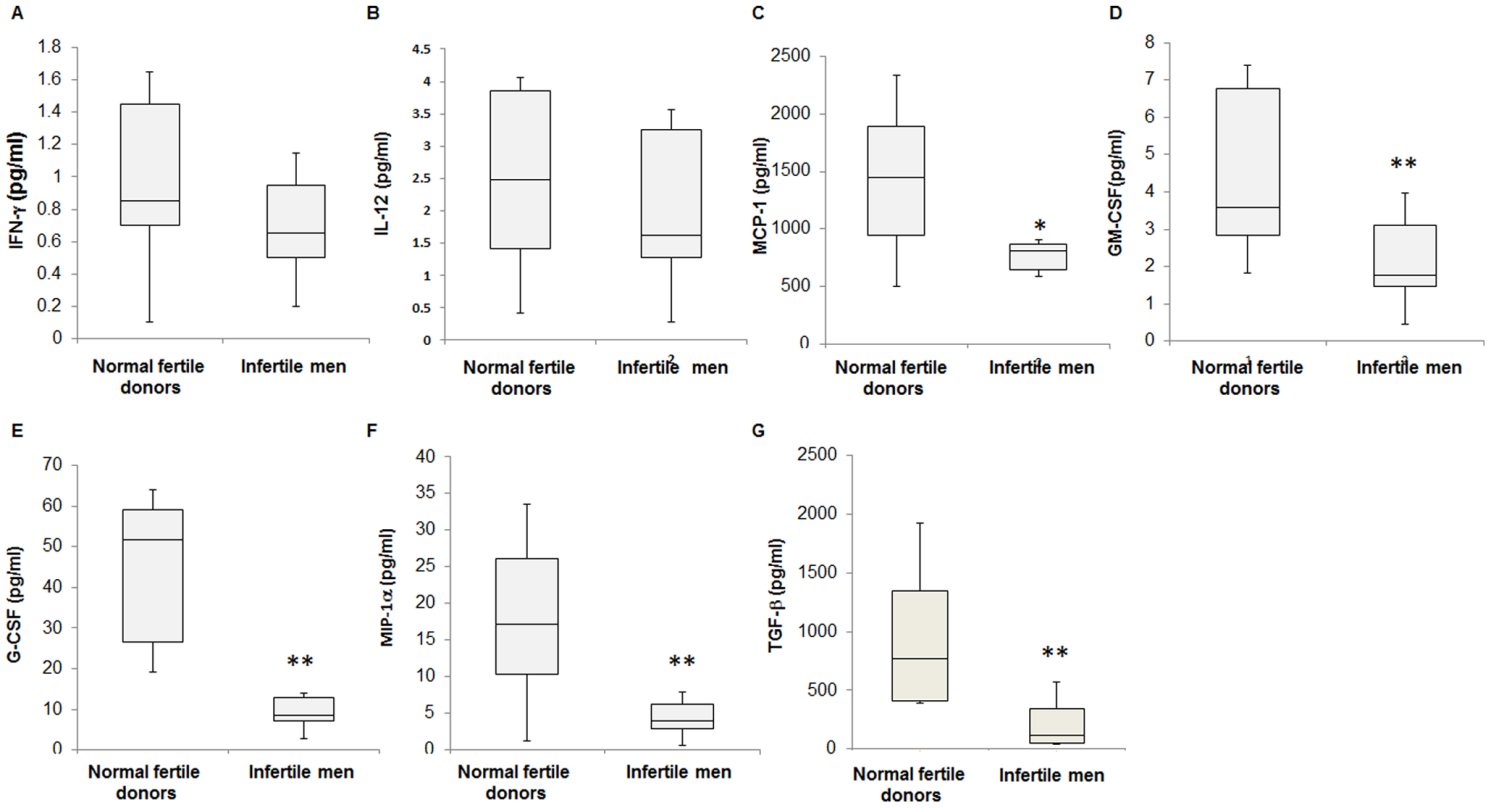
The cytokine and chemokine levels were measured by in the luminex assay and ELISA in the spermatozoa-free seminal fluid from infertile men and normal fertile donors (n = 20 each). The levels of IFN-γ (A) and IL-12 (B) were not significantly different between normal fertile donors and infertile men. The levels of MCP-1 (C), GM-CSF (D), G-CSF (E), MIP-1α (F) and TGF-β (G) were significantly decreased in infertile men compared to normal fertile donors. *P<0.05; **P<0.01 with respect to normal fertile donors.

The concentrations of MCP-1 (P<0.05), GM-CSF (P<0.01), G-CSF (P<0.01), MIP-1α (P<0.01) and TGF-β1 (P<0.01) was significantly decreased in the spermatozoa-free seminal fluid recovered from infertile men group when compared to the normal fertile donors group. In contrast, there were no significant differences in IFN-γ and IL-12 between spermatozoa-free seminal fluid from both studied groups (Fig. 2). TNF-α, IL-1β, IL-2, IL-4, IL-6 and IL-10 could not be detected in spermatozoa free-seminal fluid since they were below the sensitivity level of the assay and presumably very low. Previously, we have shown a2NTD induces the secretion of various chemokine and cytokines in the female reproductive tract [7]. To study whether secreted a2NTD of seminal fluid is correlated with secretion of various cytokines and chemokines, linear correlation analysis was performed. Significantly positive correlations were observed between the level of a2NTD and G-CSF (P<0.01), GM-CSF (P<0.01), MCP-1 (P<0.05) MIP-1α (P<0.01) and TGF-β1 (P<0.01) in spermatozoa free-seminal fluid (Table 2).

**Table 2:** Correlation between seminal fluid a2NTD, cytokine and chemokine levels (n = 40).

Cytokine/chemokine	*r* a	*P*
IL-12	0.126	N.S.
IFN-γ	0.213	N.S.
G-CSF	0.783	P<0.01
GM-CSF	0.569	P<0.01
MCP-1	0.529	P<0.05
MIP-1α	0.544	P<0.01
TGF-β1	0.607	P<0.01

a Spearman rank-correlation coefficient.

### The Expression Levels and Immunolocalization of Atp6v0a2 Protein in Immotile and Motile Spermatozoa Collected from Normal Fertile Donor

**Figure 3:**
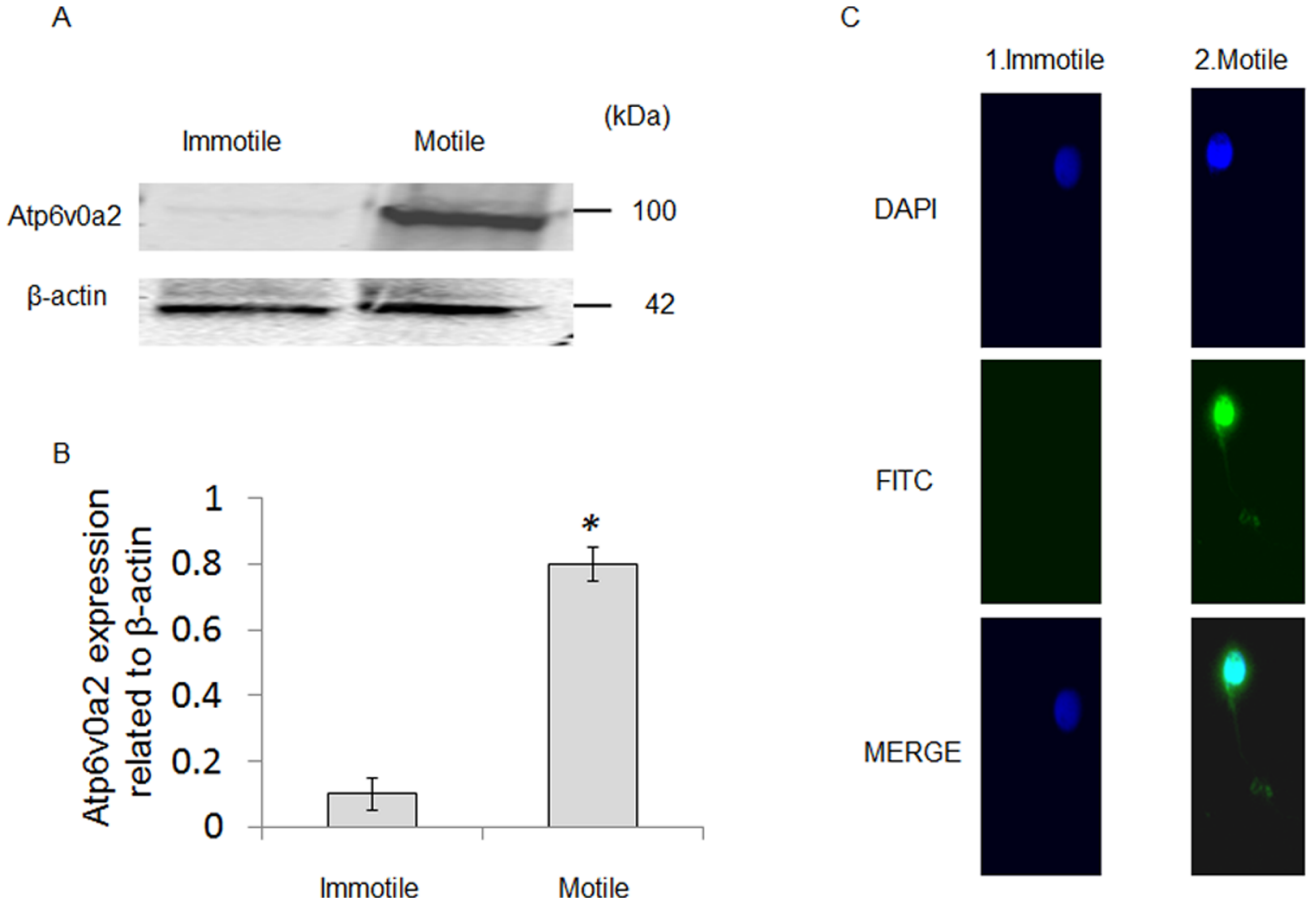
Atp6v0a2 is lower in immotile spermatozoa. (A) Western blot analysis shows the levels of Atp6v0a2 in human motile and immotile spermatozoa. (B) The bar diagram shows the quantification of Atp6v0a2 protein expression, as determined by densitometry analysis and normalized to β-actin. Immotile spermatozoa had significantly lower Atp6v0a2 expression than motile spermatozoa (P<0.01, n = 6) (*P<0.01 with respect to normal fertile donors). (C) Immunolocalization of Atp6v0a2 protein in immotile and motile spermatozoa. FITC-stained with anti-a2V antibodies (green) and DAPI nuclear stain (blue) are shown. Panel 1 indicates no expression of Atp6v0a2 protein in immotile spermatozoa. Panel 2 indicates high expression of Atp6v0a2 protein in the acrosomal region of motile spermatozoa. Data are representatives of five experiments.

To determine the association of Atp6v0a2 protein with the sperm motility, Atp6v0a2 protein level was checked in the motile and immotile spermatozoa. Western blot analysis shows that the expression of Atp6v0a2 protein was significantly higher (P<0.01; n = 6 each) in motile spermatozoa compared to immotile spermatozoa from normal fertile donors (Fig. 3A and 3B). These results suggest that the increased Atp6v0a2 protein was associated with motility of human spermatozoa.

Immunohistochemistry analysis was performed to examine the expression and localization of Atp6v0a2 protein in motile and immotile spermatozoa from normal controls. Atp6v0a2 protein was not detected in immotile spermatozoa (Fig. 3C; panel 1) however, in motile spermatozoa Atp6v0a2 protein was abundantly detected and localized in the acrosomal region (Fig. 3C; panel 2).

## Discussion

Spermatozoa deliver not only the male genome to the ova but several crucial molecules such as mRNA and proteins which are required for fertilization and early embryonic development [22], [23], [24]. Our previous studies suggested a possibility that Atp6v0a2 in spermatozoa may have an important role in fertilization [7]. In this study, we demonstrate that Atp6v0a2 is highly expressed in normal human spermatozoa but only weakly expressed in spermatozoa from infertile men. Additionally, Atp6v0a2 was highly expressed in motile spermatozoa but not immotile spermatozoa when normal spermatozoa were used. These findings suggest that Atp6v0a2 in spermatozoa plays an important role in sperm motility and is crucial for successful pregnancy.

Recently, it was shown that V-ATPases at the plasma membrane regulate the pH of intracellular organelles which activate spermatozoa motility [25] and is solely responsible for the acrosomal-region-acidification [6]. It is possible that Atp6v0a2 could play a role in spermatogenesis as pH-sensor [26]. The present results clearly indicated that Atp6v0a2 is more abundantly expressed in motile spermatozoa than in viable immotile spermatozoa. Similar to the study of Jaiswal *etal*., which investigated murine capacitated spermatozoa, Atp6v0a2 was predominantly localized in the acrosomal region of human normal spermatozoa. Atp6v0a2 is likely to be involved as a pH modulator in motile spermatozoa. Furthermore, we reported that Atp6v0a2 plays a role in the successful implantation of a murine embryo. Previously, we found that the expression of Atp6v0a2 is remarkably greater in the egg fertilized by spermatozoa than the unfertilized egg [7]. Therefore, it is speculated that Atp6v0a2 in human spermatozoa may be a vital molecule not only controlling intracellular pH, but also initiating embryogenesis.

There is an early inflammatory stage followed by an anti-inflammatory stage and both are required to for a successful pregnancy [27]. Previously, we reported that capacitated spermatozoa initiates inflammation in the maternal side by the release of the immune regulatory molecule a2NTD, which in turn induces the expression of inflammatory cytokines such as LIF, IL-1β, and TNF-α [7]. In other studies, we showed that the key portion of the a2V protein, a2NTD (the N-terminal portion) is released from the activated monocyte and induces the cytokine secretion [15]. In this study, we showed that seminal fluid from normal fertile donors was enriched with secreted a2NTD and the level of a2NTD was significantly correlated with the levels of G-CSF, GM-CSF, MCP-1, MIP-1α and TGF-β. The strongest correlation was found between a2NTD and G-CSF in seminal fluid (positive correlation coefficient of 0.784) (Table 2).

Chemokines are a family of more than thirty chemo-attractant cytokines involved in leukocyte migration, angiogenesis and cell activation. They play important roles in events associated with inﬂammation and immune defense [28], [29], and probably have similar roles in the male reproductive tract. MCP-1, which recruits monocytes, memory T-cells, and dendritic cells to sites of tissue injury, infection, and inflammation [30], [31], can control Th2 polarization to maintain the maternal immune tolerance toward the allogeneic fetus [32], [33]. Previously, we have reported that MCP-1 can induce expression of uterine Atp6v0a2 in animal model [7]. In this study, we demonstrated that MCP-1 in seminal fluid from normal fertile donors was significantly increased compared to seminal fluid from infertile men as well as a2NTD. Thus, it is possible that MCP-1 and a2NTD have a synergetic effect to establish immunological milieu at the implantation site, though further research is necessary to determine their roles in maternal–fetal tolerance.

Over the past 20 years, it has been convincingly demonstrated that the family of colony-stimulating factors (CSF) plays a pivotal role in reproduction by modulating immune milieu of reproductive tract [34], [35]. Hence, we speculate that the family of CSFs in seminal fluid may have an important role in successful fertilization. In fact, G-CSF has been reported to impair human trophoblast cell growth and function *in vitro* by inducing placental granulocytosis, and cause abortion in murine model [36]. In addition, Robertson *et al.,* reported that GM-CSF is a key factor regulating fertility and targets both dendritic cells in reproductive tract and developing embryos [35]. In this study, seminal fluid from normal fertile donors was characterized by higher levels of G-CSF and GM-CSF as compared with seminal fluid from infertile men, suggesting a yet undefined role of those. Therefore, these findings raises possibility that a2NTD in seminal fluid is associated with contribution to the establishment of early inflammatory phase which is crucial for the successful pregnancy as well as G-CSF and GM-CSF.

TGF-β is an important signaling cytokine in seminal fluid as it is well-known for its immunedeviating properties [37], [38] and plays a key role in preventing a negative immune response to spermatozoa in the female reproductive tract [39], [40]. In addition, Loras *et al.,* reported that TGF-β1 concentration was related to infertility [41]. In this study, we show that the level of TGF-β in spermatozoa free-seminal fluid from infertile men was significantly lower than those from normal fertile donors. There was a strong positive correlation coefficient between a2NTD and TGF-β1 (*r* = 0.602). It may be speculated that seminal fluid TGF-β1 is essential for initiation of immune tolerance to seminal antigens and preventing aberrant immunity to spermatozoa. These proteins appear to play a vital role in priming of a specific female immune response which is necessary for successful embryo implantation.

In this study, we analyzed whether Atp6v0a2 is associated with spermatozoa status diagnosed with the WHO semen analysis and showed that lower Atp6v0a2 expression in spermatozoa from infertile men was consistent with “abnormal” based on WHO semen analysis (i.e., parameters were lower than the standards indicated for semen volume, number and motility and morphology) [42]. With regard to spermatozoa motility, we found that Atp6v0a2 in motile spermatozoa was expressed at higher levels than in immotile spermatozoa. This result strongly suggested that Atp6v0a2 is potentially useful for the evaluation of spermatozoa motility whether semen is diagnosed normal or abnormal based on WHO semen analysis standards.

In summary, Atp6v0a2 protein and mRNA in spermatozoa from infertile men was significantly lower compared to that of normal spermatozoa. Fluorescent microscopy revealed that higher expression of Atp6v0a2 mRNA and protein in the acrosomal region of motile spermatozoa than immotile spermatozoa. Secreted a2NTD in seminal fluid from normal fertile donors was significantly higher than infertile men. Furthermore, a2NTD expression correlated with levels of cytokines and chemokines which are key molecules during the onset of pregnancy. In conclusion, a critical level of Atp6v0a2 may be necessary for the normal function of spermatozoa and decreased levels of Atp6v0a2 may predict male infertility.
